# Simulation-based training in ultrasound-guided pediatric central venous catheterization for anesthesiology residents: transfer to the clinical setting

**DOI:** 10.1186/s41077-026-00428-9

**Published:** 2026-03-05

**Authors:** M. P. Bravo, S. Silva, V. Contreras, K. Azagra, D. Barra, M. Corvetto

**Affiliations:** 1 División de Anestesiología, Escuela de Medicina, Pontificia Universidad Católica de Chile, Marcoleta 377, Santiago, 8330024 Chile; 2 Experimental Surgery and Simulation Center, Facultad de Medicina, Pontificia Universidad Católica de Chile, Santiago, Chile; 3 Departamento del Adulto, Escuela de Enfermería, Pontificia Universidad Católica de Chile, Santiago, Chile; 4 Red de Salud UC Christus, Santiago, Chile

**Keywords:** Simulation training, Anesthesiology/education, Central venous catheter

## Abstract

**Introduction:**

Simulation-based training (SBT) for central venous catheter (CVC) placement has been shown to improve procedural performance, reduce the number of attempts, and increase success rates. However, training for specific populations, such as pediatric patients, remains challenging. Moreover, evidence regarding the transfer of simulation-acquired skills to real clinical practice is limited. The aim of this study was to evaluate the transfer of skills acquired by anesthesiology residents following SBT in ultrasound-guided pediatric CVC placement.

**Methods:**

After institutional ethics committee approval, 21 anesthesiology residents were recruited to participate in an SBT program for ultrasound-guided pediatric CVC insertion. The training consisted of six individual one-hour weekly sessions with direct expert feedback, using a pediatric-specific Blue Phantom® simulation model. Pre-and post-training assessments (PRE and POST) were conducted using video-recorded procedures and evaluated by two blinded assessors using a validated Global Rating Scale (GRS). In addition, hand motion metrics were collected using the Imperial College Surgical Assessment Device (ICSAD), including total path length (TPL), number of movements (NM), and total procedure time (TPT). To assess skill transfer, the same evaluation protocol was applied during CVC insertion in pediatric patients undergoing elective cardiac surgery.

**Results:**

Fifteen residents completed the training and both PRE and POST assessments. Median GRS scores improved significantly from 34 (interquartile range [IQR], 29.5–38.0) at PRE to 47 (IQR, 44.5–47.5) at POST. Total procedure time decreased significantly from 349.4 (IQR, 264–536) to 290.2 (IQR, 232–335) seconds. No significant differences were observed in total path length or number of movements. Regarding transfer to clinical practice, 13 of 15 residents successfully completed the procedure on their first attempt in real patients. Comparison between the POST simulation assessment and the real-patient assessment showed no significant differences in median GRS scores.

**Conclusions:**

Simulation-based training significantly improved anesthesiology residents’ performance in simulated ultrasound-guided pediatric CVC placement. Although not all residents were able to successfully complete the procedure in real patients, the findings suggest a potential transfer of skills acquired through simulation to clinical practice.

**Supplementary Information:**

The online version contains supplementary material available at 10.1186/s41077-026-00428-9.

## Introduction

Simulation-based training (SBT) for central venous catheter (CVC) placement has been shown to enhance procedural competence [1, 2]. A systematic review and meta-analysis designed to assess effect of simulation training for central venous access upon procedural success in real patients reported that, compared with traditional training, simulation had a significantly larger proportion of trainees who successfully placed CVCs and fewer mean attempts to CVC insertion [3]. Recent evidence further suggests that SBT in ultrasound-guided CVC placement, focused on essential skills, such as needle and probe manipulation, reduces the incidence of mechanical complications in clinical practice [4]. Despite these advances, significant challenges remain in training for specific patient populations, particularly pediatric patients [5].

Central venous catheter insertion in infants and young children poses unique challenges related to anatomical and physiological differences compared with adults, including a relatively larger head-to-body ratio, increased collapsibility of central veins, smaller vessel caliber, and limited anatomical landmarks. These factors increase procedural complexity and the risk of complications.

The development of simulation-based ultrasound-guided CVC training programs tailored to pediatric patients is therefore essential. Pediatric CVC placement is associated with potentially serious complications, including injury to adjacent structures and catheter-associated bloodstream infection [6]. Moreover, training opportunities in pediatric populations—particularly for residents—are inherently limited, as this procedure is performed infrequently in routine clinical practice. A structured SBT program would be highly relevant across multiple residency programs, including anesthesiology, emergency medicine, intensive care, and pediatric surgery.

An additional challenge in designing SBT programs is determining whether skills acquired in the simulation environment can be effectively transferred to clinical practice with real pediatric patients. Transfer of learning remains an area of active investigation and ongoing debate [7, 8]. Several factors have been identified as influencing skill transfer, including the type of skill being trained, the fidelity and realism of the simulation environment, alignment between simulated and clinical contexts, and learner characteristics [9]. However, robust evidence remains limited, particularly regarding short- and long-term transfer and the impact of simulation-based training on specific pediatric patient groups.

In this context, the primary objective of this study was to develop a simulation-based training program for ultrasound-guided CVC placement in pediatric patients (ranging from toddlers to preschool-aged children) and to evaluate the transfer of skills acquired by anesthesiology residents to the clinical setting.

## Methods

### Study design

This prospective quasi-experimental pre–post study evaluated a simulation-based training program designed to develop proficiency in ultrasound-guided pediatric central venous catheter (CVC) insertion, conducted in accordance with guidelines for healthcare simulation research [10].

### Participants

The institutional ethics committee approved this study and required informed consent (approval number 220503003; Comité Ético Científico de Ciencias de la Salud UC, Facultad de Medicina, Pontificia Universidad Católica de Chile). The study was conducted in accordance with the Declaration of Helsinki.

Twenty-one second- and third-year anesthesiology residents were invited to participate in the protocol. Residents were all part of a university residency training program at Pontificia Universidad Católica de Chile. Participation was voluntary, and no exclusion criteria were applied regarding prior experience or previous ultrasound training.

### Educational support material

Educational materials covering both theoretical and practical aspects of ultrasound-guided techniques and central venous catheterization were provided through an online platform. Residents were instructed to review these materials prior to the start of the protocol, including assessments and training sessions, to ensure a homogeneous theoretical baseline among participants. The instructional content was accessible at any time and from any location via the online platform, using personal electronic devices with internet access throughout the study period. The material was progressively released as participants advanced. Upon completion, the platform provides confirmation that the resident had reviewed all readings and videos. This confirmation was mandatory prior to initiating the training.

### Intervention

Simulation-based training comprised six individual sessions conducted at the Simulation Center, each lasting one hour and scheduled on a weekly basis. Residents participated in one-to-one practice sessions with an instructor. Training was supervised by two faculty members with extensive experience in simulation-based education and infant vascular access, both practicing congenital cardiac anesthesia.

Based on the previous experience of our simulation group, we created a simulation-based program to train our residents in CVC placement in infants through internal jugular vein access [1, 9]. This program was designed using a competency-based training approach built on principles of deliberate practice, including the disaggregation of skills identified by prior process mining analysis. The access chosen was the right internal jugular vein, a common cannulation site for infants undergoing cardiac surgery at our institution. To create the instructional model for simulated sessions, a critical literature review was done. An analysis based on process mining analysis of CVC insertion procedure was used, to identify and pinpoint more difficult steps [11–13]. Process mining is an emerging discipline that enables the systematic analysis of procedural executions within training environments. It provides objective insights into adherence to a predefined normative procedural model (i.e., process similarity), the frequency of step repetitions or reworks, and additional performance metrics. These data can be leveraged to deliver structured, objective feedback to trainees, thereby supporting a process-oriented feedback approach that facilitates deliberate practice and targeted skill development. The conclusions of this analysis are summarized here:

- Train partial tasks and then integrate an ordered sequence of these tasks into the complete process [11].
- Ultrasound guided approach and real-time puncture (dynamic tracking of the needle tip) [14, 15]. A 22G peripheral IV catheter was used.
- Guidewire insertion with a fixed tripod type hand [12].
- Anticipate common clinical errors in the pediatric population: Familiarize with ultrasound images of vessel transfixion and train how to rescue the puncture (a common scenario in small and collapsible vessels). Given the difficulty of dilation, the correct use of the scalpel to widen the skin entry site and control the dilator advancement.
- Define safety standards for the procedure using ultrasound: Identify the vein’s anatomy and patency, perform real-time ultrasound-guided puncture, confirm the peripheral IV catheter position and then guidewire within the vein prior to dilatation [16]. Failure to perform this last step was considered as a technique failure. If this step is omitted, the entire procedure must be restarted from the beginning.

**Fig. 1:**
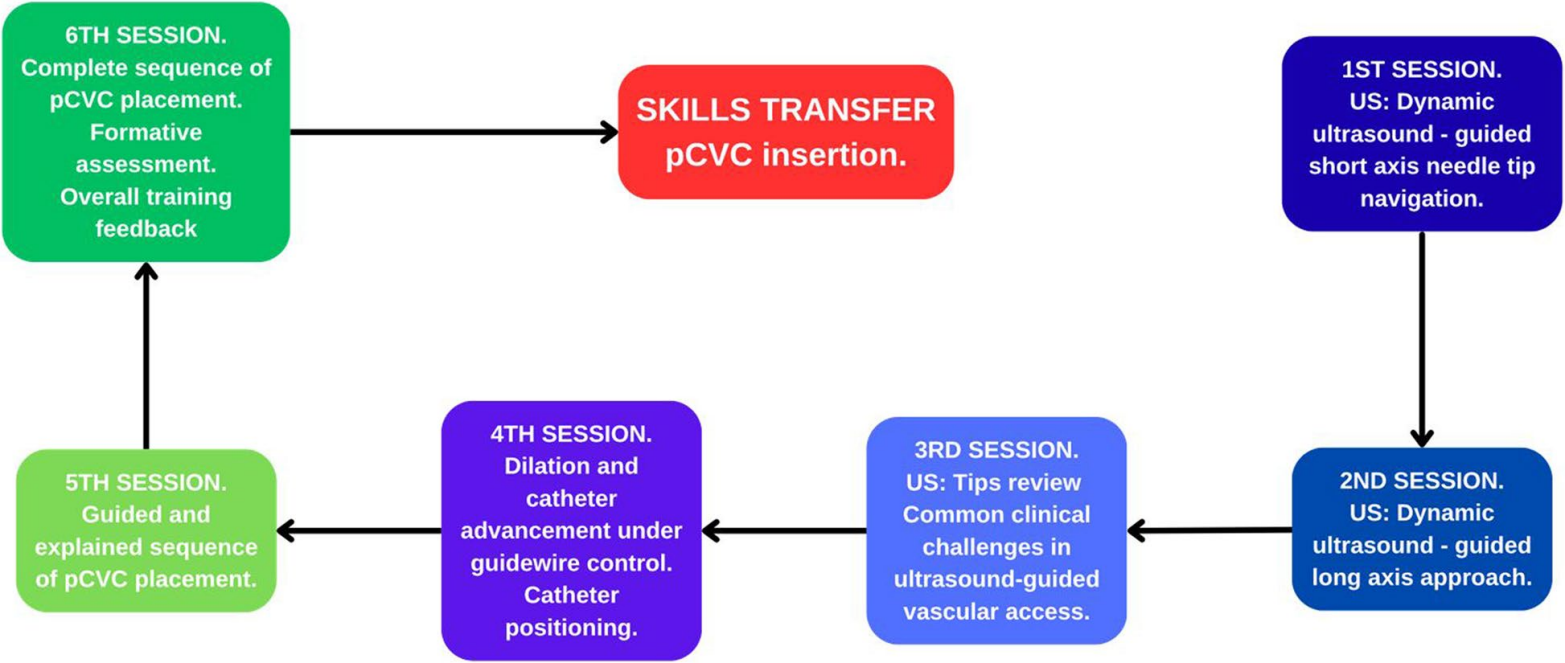
Roadmap with specific goals for the practical sessions 1 to 6

During the six-session training program, participants practiced ultrasound scanning and vascular puncture skills required for pediatric central venous catheter (CVC) insertion. The first four sessions focused on partial-task training, whereas the final two sessions emphasized the integration of these component skills into the complete procedural sequence. A standardized roadmap outlining specific objectives for each practical session was provided to instructors to facilitate consistent and structured instruction (Fig. 1).

- Session 1: Dynamic needle tip positioning (different vessel sizes).
- Session 2: Long-axis puncture (perpendicular and oblique probe).
- Session 3: Common clinical tips and problems.
- Session 4: Dilation and controlled guidewire advancement.
- Session 5: Complete insertion sequence explained (clinical tips) and on-site feedback.
- Session 6: Complete sequence/formative assessment/feedback.

Each session began with a warm-up phase to assess retention of the skills acquired in the previous session before progressing to the next objective. Instructors then outlined the specific goals of the session, followed by focused skill practice with concurrent feedback.

**Fig. 2:**
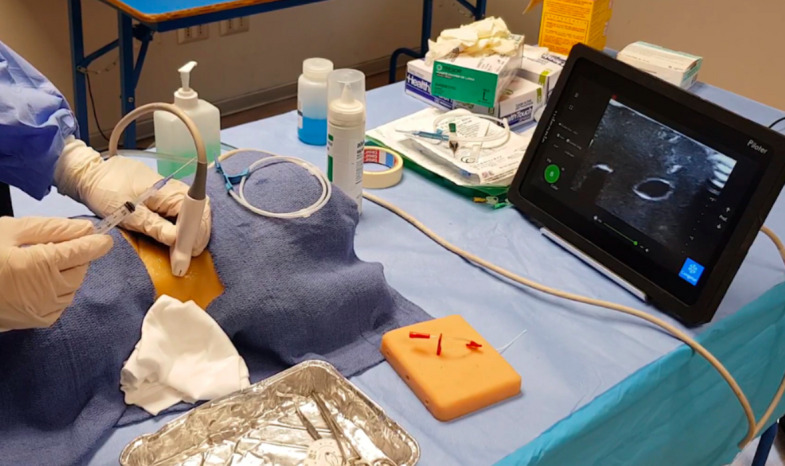
Resident in the simulation training station

**Fig. 3:**
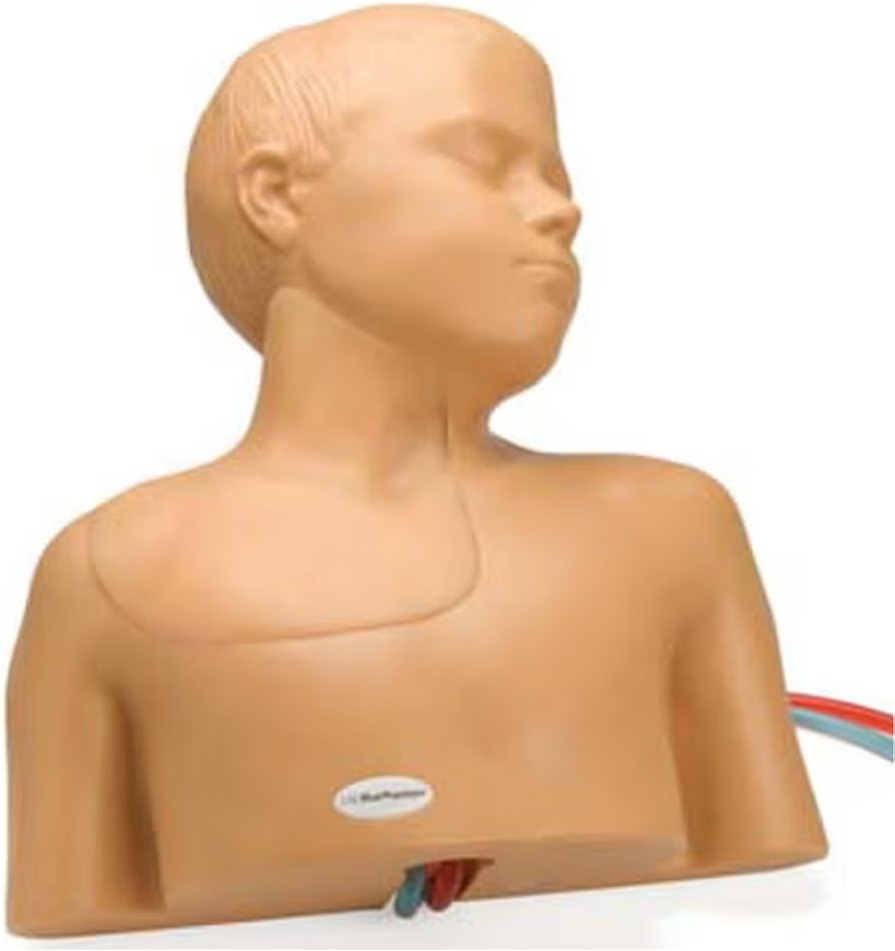
Pediatric central line ultrasound training model (Blue Phantom®, Redmond, WA)

The instructor was present throughout the entire session and provided real-time guidance and direct feedback using a traditional face-to-face feedback approach. Training was conducted using a branched two-vessel ultrasound training block (Blue Phantom®, Redmond, WA) and a dedicated pediatric central venous catheter ultrasound training model (Blue Phantom®, Redmond, WA) (Figs. 2 and 3).

### Assessments

Following completion of the educational support materials and prior to initiation of training sessions, residents underwent a pre-intervention assessment (PRE), which consisted of performing an ultrasound-guided pediatric central venous catheter insertion using previously described part-task trainer. Upon completion of the program, residents underwent a post-training assessment (POST).

Both PRE and POST assessments were video recorded by simulation center staff using a standardized setup to capture the participant’s hand movements and the ultrasound image. The recorded videos were anonymized, pooled across PRE and POST assessments, and limited to views of the operator’s hands. Two independent, blinded reviewers evaluated each resident’s performance using the previously described Global Rating Scale (GRS) [17]. The GRS was evaluated within the context of a formative examination using simulation in the assessment of CVC skills, with better results than two published available checklists. The GRS assessed general procedural behaviors, including time and motion efficiency and procedural flow, and consisted of nine items rated on behaviorally anchored 5- or 6-point scales, yielding a maximum total score of 50 (Appendix 1). Reviewers were not involved in the instructional component of the training.

During the assessments, two sensors from a motion-tracking device were attached to the operator’s hands. The Imperial College Surgical Assessment Device (ICSAD) tracks hand movements during procedural tasks using sensors placed on the dorsum of both hands [18]. The total path length of both hands serves as an objective indicator of technical skill and movement efficiency during manual tasks. In this study, the ICSAD recorded total path length (TPL), number of movements (NM), and total procedure time (TPT). Simulation center staff were responsible for sensor placement and data acquisition.

Upon completion of the six-session training program, the same assessment was conducted in a clinical setting to evaluate the transfer of skills acquired in the simulation environment to real patients. Residents performed an ultrasound-guided central venous catheter insertion in a pediatric patient (ranging from toddlers to preschool-aged children) prior to elective cardiac surgery. A staff anesthesiologist responsible for the case was present with the resident in the preanesthesia care unit to ensure patient safety and adherence to the study protocol. Residents were allowed a single attempt to complete the procedure and were permitted to terminate the procedure if deemed necessary. No feedback was provided during the assessment.

ICSAD sensors were placed on the resident’s hands beneath surgical gloves to maintain sterile technique. The procedure was video recorded using the same standardized methodology described above, capturing the operator’s hand movements and the ultrasound image. Simulation center personnel were responsible for ICSAD setup and video recording. Subsequently, two independent reviewers evaluated resident performance using the same Global Rating Scale (GRS). All videos were anonymized and limited to views of the operator’s hands and ultrasound image.

Finally, an online satisfaction survey was administered to residents to assess their perceptions upon completion of the training program [19]. Satisfaction survey consisted in a questionnaire with 5-item Likert questions, around the achievement of objectives, educational material, duration of training, quality of instructors’ feedback and satisfaction with the simulation-based training program.

### Sample size

An a priori sample size calculation determined that 11 participants were required to detect a difference in the primary outcome (Global Rating Scale [GRS] score). The calculation assumed an effect size of 1.5 (Cohen’s *d*), a statistical power of 0.80, and a paired-sample design comparing pre- and post-training assessments. The assumed effect size was derived from previously published studies evaluating improvements in GRS scores following simulation-based training for ultrasound-guided central venous catheter insertion [2].

### Statistical analysis

Data were analyzed using SPSS software (version 15.0; Chicago, IL, USA) and JASP software (version 0.19.1). A nonparametric distribution of the data was assumed. Global Rating Scale (GRS) and Imperial College Surgical Assessment Device (ICSAD) outcomes were summarized as medians with interquartile ranges. Pre- and post-training comparisons were performed using the Wilcoxon signed-rank test. Interobserver agreement was assessed by calculating the intraclass correlation coefficient (ICC). A p-value of 0.05 was considered significant.

## Results

**Fig. 4:**
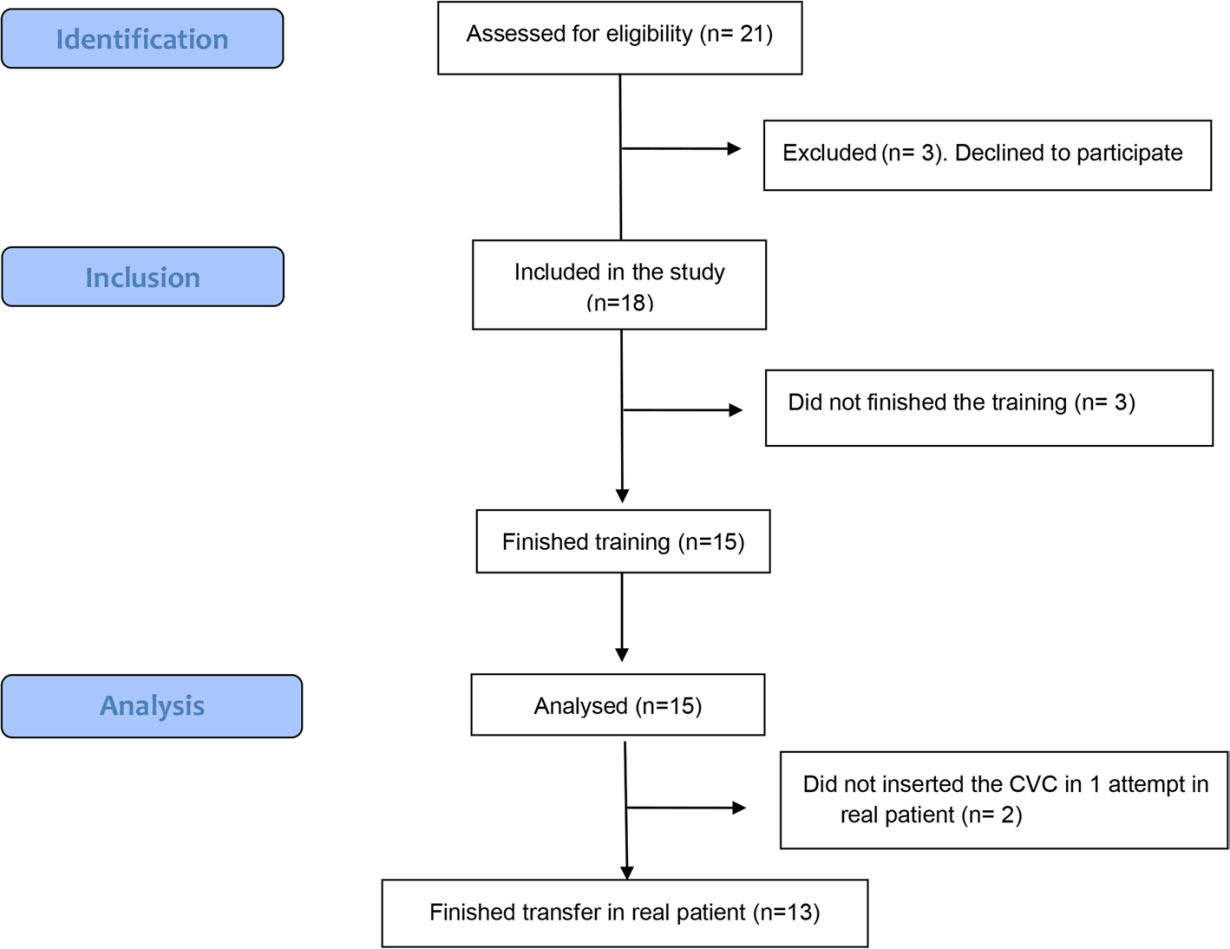
Median value and interquartile ranges of Global Rating Scale (GRS) score of residents during pre-assessment, post-assessment and the transfer session

Fifteen residents completed the six-session training program and all scheduled assessments. Of the 21 residents invited to participate, three declined enrollment. Among the 18 residents who were enrolled, three did not complete the training within the predefined study period because of personal reasons, resulting in a dropout rate of 16.7%. The flow of participants through the study is illustrated in Fig. 4.

Demographic characteristics, as well as prior ultrasound training and central venous catheter (CVC) experience, are summarized in Table 1. All participants were second- or third-year anesthesiology residents. Overall, residents reported limited prior experience with pediatric CVC insertion, and none had previously received simulation-based ultrasound training.

**Table 1:** Demographic data (*n* = 15)

	**N (%) or Median (IQR)**
Age	30 (25–34)
Sex	
Male	8 (40)
Female	7 (60)
Year of Residency	
PGY-2	11 (73.3)
PGY-3	4 (26.7)
Previous pediatric CVC	
0	9 (60)
1–3	4 (24)
> 3	2 (16)
Previous SBT	
Intravenous Access	2 (13)
Central Venous Catheter	0 (0)
PNB	6 (40)

*IQR* Interquartile Range, *CVC* Central venous catheter, *SBT* Simulation-based Training, *PNB* Peripheral Nerve Block

**Fig. 5:**
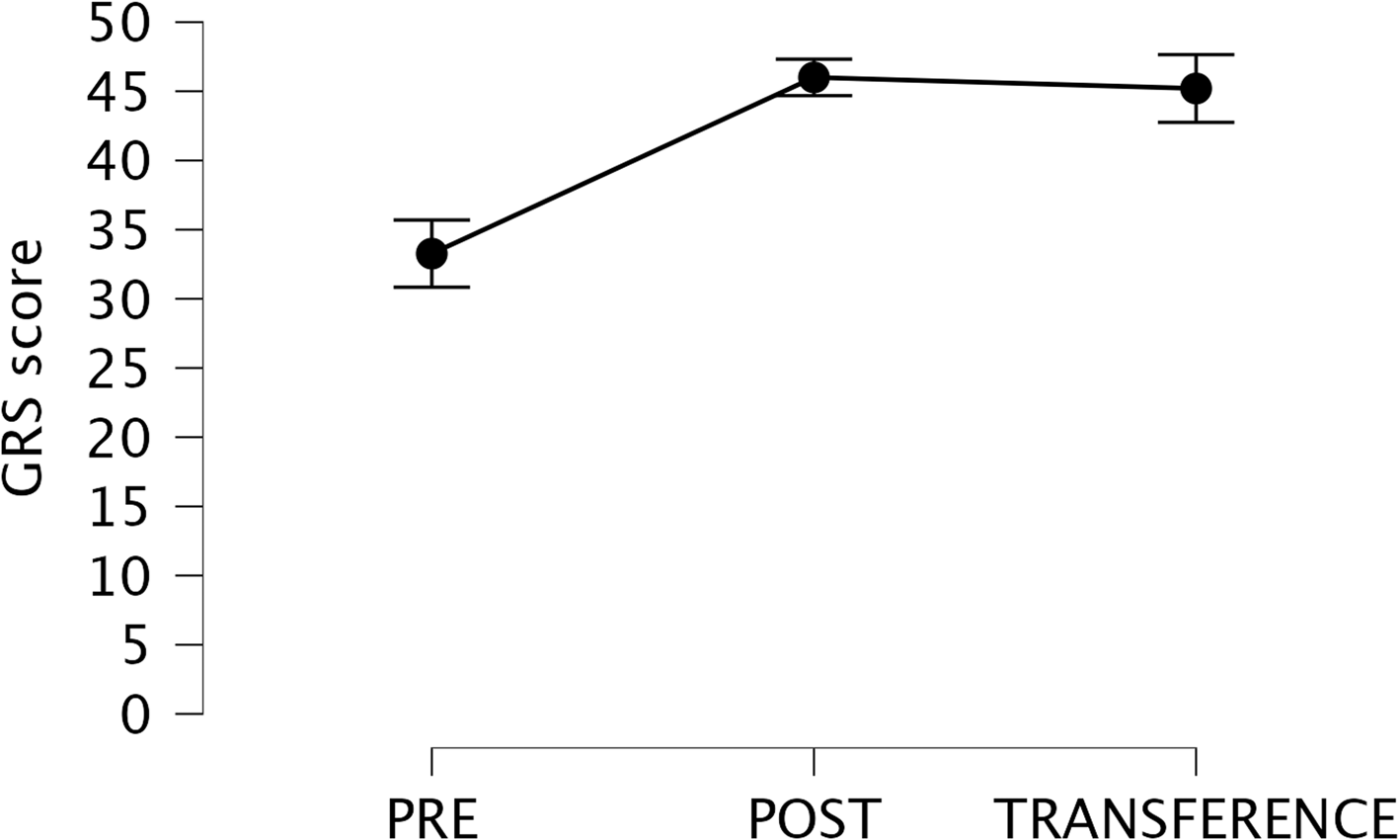
Participants’ flow diagram

Inter-rater reliability for the observers’ video-based Global Rating Scale (GRS) assessments demonstrated good agreement, with an intraclass correlation coefficient (ICC) of 0.76. Median GRS scores increased significantly from 34 (interquartile range [IQR], 29.5–38.0) at baseline to 47 (IQR, 44.5–47.5) after training (*p* < 0.001) (Table 2). Figure 5 illustrates the progression of individual residents’ GRS scores from the pre-training (PRE) to the post-training (POST) assessment.

**Table 2:** Comparison between PRE and POST assessments

	**PRE**	**POST**	***P* value**
GRS scores	34 (29.5–38.0)	47 (44.5–47.5)	< 0.001
TPL (m)	60.32 (47–80)	60 (43–84)	0.570
NM	301 (263–443)	298 (245–468)	0.561
TPT (s)	349.4 (264–536)	290.2 (232–335)	0.026

*GRS* Global rating scale scores, *TPT* Total procedural time, *NM* Number of movements, *TPL* Total path length

Data are summarized as median and interquartile range

*P* values obtained when comparing with Wilcoxon signed-rank test

*P* value is considered statistically significant when < 0.05

Outcomes measured using the Imperial College Surgical Assessment Device (ICSAD), including total path length (TPL), total procedure time (TPT), and number of movements (NM), are presented in Table 2. A significant reduction in TPT was observed, decreasing from 349 to 290 s (*p* = 0.026). Although improvements were observed in TPL (from 11.07 to 9.4 m; *p* = 0.322) and NM (from 48 to 41; *p* = 0.262), these changes did not reach statistical significance.

With respect to transfer of skills to the clinical setting, 13 residents successfully completed the procedure on their first attempt in the operating room. Successful performance was defined as achieving venous cannulation on the first attempt without immediate complications, including arterial puncture, hematoma formation, or arrhythmias. The pediatric patients had a mean weight of 9.7 kg (range, 5–20 kg), and all were scheduled for elective cardiac surgery.

When comparing post-training (POST) simulation assessments with subsequent performance in real patients, no significant differences were observed in median Global Rating Scale (GRS) scores or total path length (TPL) values (Table 3). However, residents required significantly more time to complete their first central venous catheter insertion in a real patient compared with the POST simulation assessment (median, 467 vs 290 s; *p* = 0.001).

**Table 3:** Comparison between POST and transference assessments

	**POST**	**Transference**	***P* value**
GRS scores	47 (44.5–47.5)	46 (43–49)	0.805
TPL (m)	60 (43–84)	99.62 (67–158)	0.33
NM	298 (245–468)	510 (357–869)	0.048
TPT (s)	290.2 (232–335)	467.1 [418–539]	0.001

*GRS* Global rating scale, *TPT* Total procedural time, *NM* Number of movements, *TPL* Total path length

Data are summarized as median and interquartile range

*P* values obtained when comparing with Wilcoxon signed-rank test

*P* value is considered statistically significant when < 0.05

Resident satisfaction with the training program was high. Residents reported that program objectives were clearly defined (100% Likert 5), instructor feedback was clear and focused on the learning objectives (100% Likert 5), and duration of training was adequate (80% Likert 5, 20% Likert 4).

## Discussion

Residents demonstrated improved performance in ultrasound-guided pediatric central venous catheter insertion following the simulation-based training. Although not all residents were able to successfully perform the procedure on real pediatric patients (ranging from toddlers to preschoolers) on their first attempt, the findings suggest a potential transfer of skills from simulation to clinical practice. The ICSAD motion-tracking device, however, did not detect significant differences in performance before and after training in this context.

Previous simulation-based training programs for CVC placement have demonstrated similar improvements in learners’ performance in a simulated setting [1–3, 20, 21]. Many simulation-based CVC placement training programs have been described, but they do not address the specific considerations for infant pediatric CVC placement. Training of CVC placement in the pediatric population may be challenging, due to anatomic factors, such as the smaller size of the vessels and the different consistency of the tissues. More recently, specific programs for ultrasound-guided pediatric CVC placement have been described [22, 23]. Good et al. implemented a simulation-based training program that increased knowledge, skill, and confidence in pediatric CVC placement for pediatric critical care medicine and pediatric emergency medicine fellows [23]. This study demonstrates an improvement in the outcome skill, reflected in the reduction of procedure time. Specifically, the median procedure time decreased from 264 to 146 s. In our study, we observed a more modest yet still significant improvement, with times decreasing from 349 to 290 s. Although the difference is approximately one-minute, procedural time represents a critical component of overall performance and clinical safety.

Regarding the assessment in the real patient situation, the concept of transfer of learning refers to the fact that skills acquired through simulation-based training may be applied by students on real patients in the clinical settings [24]. Simulation-based training is effective when learners are prepared to apply what they learned in the simulated laboratory to real patients in the clinical setting [25]. This corresponds to the third level of Kirkpatrick’s model, “behavior”. Few simulation-based training studies have put emphasis on this outcome, and most studies assess Kirkpatrick levels 1 or 2, satisfaction, knowledge and skill in a simulation environment [26–28]. Barsuk shown an increase in residents’ skills in simulated CVC insertion after a simulation-based program and later a decreased the number of needle passes when performing actual procedures in a medical intensive care unit [20]. Residents were surveyed regarding the number of needle passes and complications; however, their procedural performance was not objectively assessed. In this context, a lack of studies assessing transfer in pediatric CVC insertion, prevent comparison of our results with previous studies. Considering the challenges of measuring CVC placements in small children, the measurements were successfully obtained in this protocol. Our results showed that 13 of the 15 residents did the procedure in the first attempt in real patients and median values of GRS scores were not different to the last simulated session. This finding may reflect the successful transfer of skills acquired in the simulated environment to performance in real clinical patients. However, residents needed significantly more time to perform the procedure in the real patient. A longer duration in the real setting may be understood as adaptive prudence in a more complex context. Transfer of CVC insertion in pediatric patients may be challenging, due to the high cognitive load of residents in a cardiac surgery ward. Additionally, controlling for patient size in this context is challenging (weight between 5 to 20 kilos). Although our institution is a cardiac surgery referral center, most procedures requiring a central venous catheter (CVC) are performed in small children. Consequently, training on a preschool-sized phantom and subsequently applying those skills to smaller patients posed an evident challenge.

Regarding the use of the tracking motion device ICSAD, this device has previously demonstrated construct and concurrent validity in labor epidural placement, spinal anesthesia, ultrasound-guided supraclavicular block, and jugular CVC placement. [29–32] Specifically for CVC placement, ICSAD was validated because total path length (TPL) discriminates between expert and novices and correlates with a previous, validated GRS [31]. A lack of power might explain this situation, because the sample size was calculated for the primary outcome (GRS scores). Another possible explanation is that, given these residents’ prior clinical experience with adult CVC placement (reflected in their baseline score of 34 points), significant changes are difficult to observe.

This study has several limitations. First, the absence of a control group not exposed to simulation-based training precludes direct comparison with standard educational approaches. In addition, the study was conducted within the context of curricular implementation, which limits the ability to infer a causal relationship between the intervention and the observed outcomes. Without a control group, it is not possible to determine the extent to which improvements in Global Rating Scale (GRS) scores can be attributed to the structured simulation-based training itself, as opposed to other factors such as natural skill maturation, concurrent clinical exposure, or test–retest effects. Second, ICSAD sensors failed to discriminate the performance improvement after this simulation-based training program. Beyond the potential issue of limited statistical power, another plausible explanation is the insufficient sensitivity of the measurement device to detect changes in a procedure characterized by very “small” spatial variations.

Finally, as noted above, the pediatric central line ultrasound training model used in this study was a preschool-aged mannequin, whereas the real patients were toddlers and preschoolers.

## Conclusion

Residents demonstrated significant improvement in simulated ultrasound-guided pediatric central venous catheter insertion following a six-session simulation-based training program grounded in deliberate practice. In most cases, these performance gains translated to clinical practice in pediatric patients, as reflected by improvements in the primary outcome measure, the Global Rating Scale (GRS). In contrast, the Imperial College Surgical Assessment Device (ICSAD) did not detect significant pre- to post-training differences in this setting.

Pediatric CVC insertion is a procedure with limited opportunities for clinical exposure, underscoring the importance of simulation-based training programs [33]. Future efforts should focus on the development of age-specific ultrasound-guided CVC simulators (e.g., newborn, infant, and toddler models) to enhance realism and facilitate more direct transfer of skills to clinical practice.

## Supplementary Information

Supplementary Material 1. Appendix 1. Global rating scale for the assessment of central venous catheterization skills using simulation

## Data Availability

The datasets used and/or analyzed during the current study are available from the corresponding author on reasonable request.

## Abbreviations

CVC: Central venous catheter
GRS: Global rating scale
ICSAD: Imperial College Surgical Assessment Device
TPL: Total path length
NM: Number of movements
TPT: Total procedure time
ICC: Intraclass correlation coefficient
SBT: Simulation-based training
