# Supplementary figures and images for: Simulation-based training in ultrasound-guided pediatric central venous catheterization for anesthesiology residents: transfer to the clinical setting
Source: Adv Simul (Lond). 2026 Mar 5;11:18. doi: 10.1186/s41077-026-00428-9 (PMC12961813; doi:10.1186/s41077-026-00428-9)

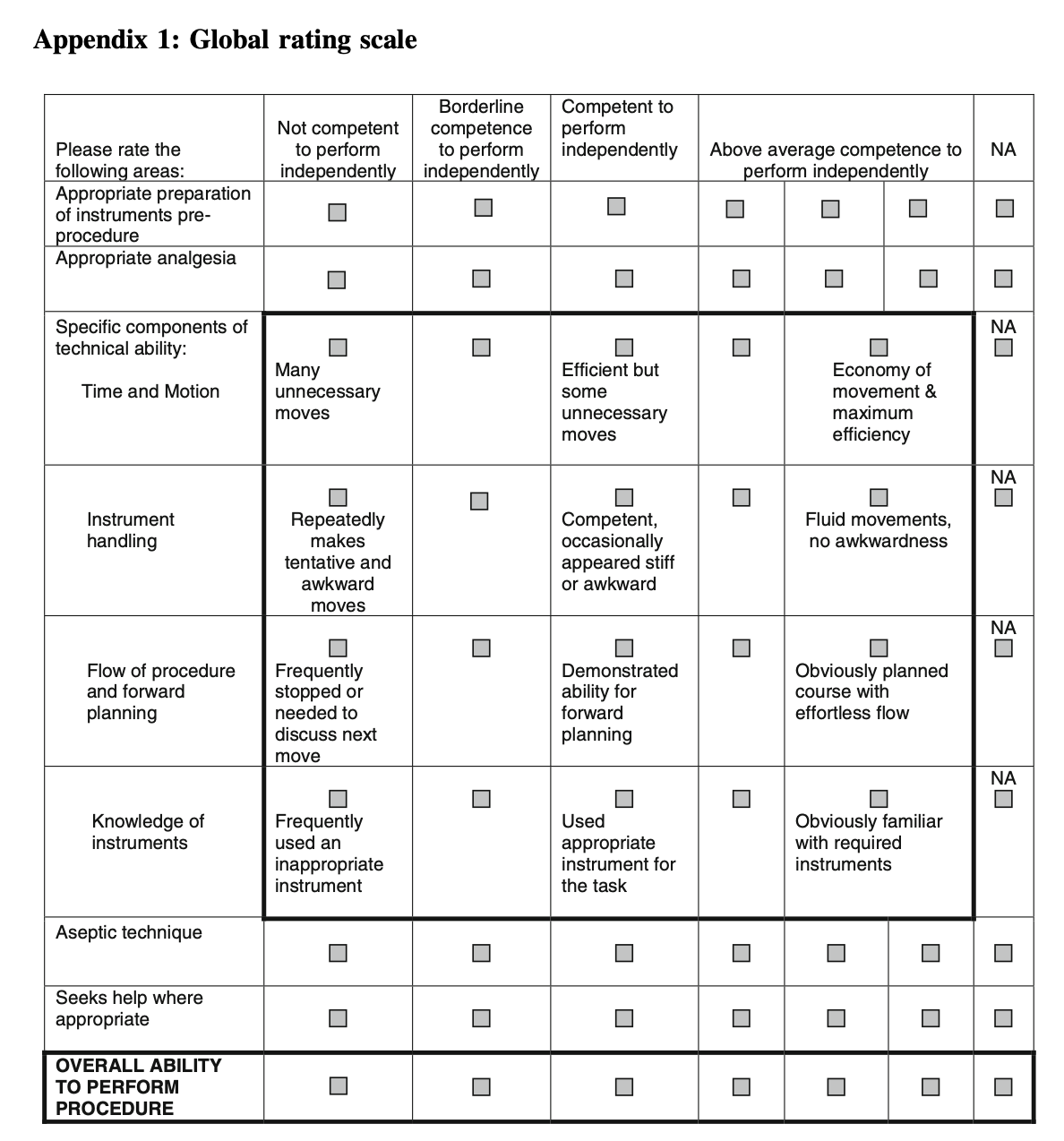

Supplement: Supplementary file 1 — Supplementary Material 1. Appendix 1. Global rating scale for the assessment of central venous catheterization skills using simulation [file 41077_2026_428_MOESM1_ESM.png]
